# Impact of Ocean Acidification on the Intestinal Microbiota of the Marine Sea Bream (*Sparus aurata* L.)

**DOI:** 10.3389/fphys.2019.01446

**Published:** 2019-11-28

**Authors:** Filomena Fonseca, Ricardo Cerqueira, Juan Fuentes

**Affiliations:** ^1^Centro de Investigação Marinha e Ambiental, Universidade do Algarve, Faro, Portugal; ^2^Centre of Marine Sciences, Universidade do Algarve, Faro, Portugal

**Keywords:** sea bream, intestinal, microbiota, ocean acidification, *Vibrio*

## Abstract

Within a scenario of increasing atmospheric CO_2_ and ocean acidification (OA), it is highly relevant to investigate its impacts not only on fish performance but also on fish intestinal microbiome and how that reflects on host performance and health. The main objective of this study was to establish if the intestinal microbiota of the sea bream (*Sparus aurata*) was affected by high level of CO_2_ in line with the predictions for this century. The bacterial communities of the intestinal fluid were characterized in animals kept at the present-day level of CO_2_ (400 μatm) and in animals switched to high CO_2_ (1200 μatm) for 1 month. Bacterial taxa identification was based on molecular methods, using the DNA coding for the 16S ribosomal RNA and primers targeting the regions V1–V3. Amplicons obtained from DNA samples of animals in the same tank were combined, cloned to obtain a bacterial DNA library, and the clones were sequenced. No significant differences were found between the two treatments for alpha diversity. However, beta diversity analysis revealed distinct dysbiosis in response to hypercapnia, with phylum Firmicutes absent from the bacterial communities of fish exposed to 1200 μatm CO_2_, whereas Proteobacteria relative abundance was increased at elevated CO_2_, due to the presence of Gammaproteobacteria (*Vibrionaceae* and *Alteromonadaceae*), a class not present in the control samples. This study provides a first glimpse at the impact of OA in fish intestinal microbiota and highlights potential downstream effects to the general condition of fishes under hypercapnia.

## Introduction

In a global scenario, as a result of anthropogenic emissions in the atmosphere, mean oceanic CO_2_ values are expected to reach 1000 μatm CO_2_ by year 2100 and 1900 μatm CO_2_ by year 2300 (Caldeira and Wickett, 2003; Meehl et al., 2007). The ocean will act as a sinkhole of this CO_2_ resulting in a decrease of pH (IPCC, 2007, 2014; Kroeker et al., 2013), equivalent to a decline of 0.32 and 0.77 units, respectively, in the average ocean surface pH (Caldeira and Wickett, 2005). This shift in ocean chemistry is expected to have a profound impact in marine organisms (Secretariat of the Convention on Biological Diversity, 2014), as has been discussed in the literature with special emphasis on fish [see the review by Heuer and Grosell (2014) and references there in].

It has been shown that at higher partial pressure of CO_2_ (*p*CO_2_), the marine fish intestine has an increased metabolic demand (Heuer and Grosell, 2016), coupled to an increased intestinal ion transport and movement of plasma HCO_3_^–^ into the intestinal lumen in exchange for Cl^–^. Compensation for elevated *p*CO_2_ typically occurs within days (Esbaugh et al., 2012) and, as a consequence, the mechanism of adaptation of marine fish to hyperosmotic environment, naturally present in the intestine, is intensified by hypercapnia. As such, an increase in HCO_3_^–^ secretion into the intestinal lumen promotes a faster increase in pH levels in parallel with the accumulation of calcium and magnesium in the intestinal fluid. This results in increased formation of carbonate aggregates, reduced osmolality and facilitated water absorption (Gregório et al., 2019) at high *p*CO_2_. The augment in energetic cost for ion transport processes and the increased O_2_ consumption observed in the intestine at elevated *p*CO_2_, may diminish the energy available for digestive functions, by slowing the process of digestion which has been observed in different species (Tirsgaard et al., 2015; Heuer and Grosell, 2016).

The gastrointestinal tract (GIT) is a favorable ecological niche for microorganisms and several reviews have highlighted the roles played by the microbial communities, strongly suggesting that fish gut microbiota is involved in energy homeostasis by affecting processes such as feeding, digestion, metabolism and also the immune response (Nayak, 2010; Egerton et al., 2018; Wang et al., 2018; Butt and Volkoff, 2019). The fish gut bacterial communities may contain pathogenic, symbiotic and commensal bacteria and they can exert great effect on the host welfare which may result in mucosal tolerance or inflammation (Gomez and Balcázar, 2008; Merrifield et al., 2010). It is well accepted that the microbiota–gut–brain axis (MGB axis) has a pivotal role in host health, stress physiology and behavior (Mueller et al., 2012; Llewellyn et al., 2014; Foster et al., 2017).

In terms of structure there is a distinction between the allochthonous, or the free-living, microbiota associated with the digesta, and the autochthonous communities, which are able to colonize the mucosal surface of the digestive tract (Nayak, 2010; Banerjee and Ray, 2017). The density, composition and function of the autochthonous microbiota communities, in contrast with the allochthonous bacteria, varies distinctly in the different sections of the fish GIT (Nayak, 2010; Clements et al., 2014; Banerjee and Ray, 2017).

The intestinal allochthonous microbiota, more abundant and variable in composition than the autochthonous communities, has been suggested to play an important role in nutrient digestion and absorption (Ramirez and Dixon, 2003; Merrifield et al., 2010; Dimitroglou et al., 2011).

The dietary effect on fish gut microbiota has received special attention in the context of aquaculture of economically important species such as seabream (de Paula Silva et al., 2011; Estruch et al., 2015; Piazzon et al., 2017; Nikouli et al., 2018), salmon (Green et al., 2013; Dehler et al., 2017; Uren Webster et al., 2018) and cod (Ringø et al., 2006; Zhou et al., 2013), to name a few. Besides dietary and host-specific intrinsic factors (genetics, age, feeding habits), environmental factors such as wild vs. rearing conditions, seasonality, salinity, and temperature have also been studied and are known to influence fish gut microbiota structure (Nayak, 2010; Tarnecki et al., 2017; Egerton et al., 2018; Butt and Volkoff, 2019).

Given the intestinal environment and functioning modifications observed at elevated CO_2_, it is reasonable to expect that these changing conditions might affect the microbiota associated with the fish gut. As such, alterations in the quality of microorganisms in the GIT, which can be reflected in the host by mutualistic relation effects, could constitute another downstream impact of CO_2_ hitherto overlooked.

However, to the best of our knowledge, how the chemical and physiological compensatory processes that take place at elevated CO_2_ interact with the intestinal microbiota are still to be analyzed. In view of this, the aim of the present work was to investigate the effect of elevated CO_2_ in the bacterial community associated with intestinal fluid of sea bream (*Sparus aurata*), using molecular methods.

## Materials and Methods

### Ethics Statement

All the procedures were approved by CCMAR Animal Welfare Committee (ORBEA CCMAR-CBMR) and Direcção-Geral de Alimentação e Veterinária (DGAV) of the Portuguese Government, according to national legislation translation of EU Directive 2010/63/EU. Procedures were only applied to live animals by authorized users.

### Animals, Experimental Conditions, and Sampling

Sea bream (*S. aurata*) juveniles were purchased from CUPIMAR SA (Cadiz, Spain) and transported to Ramalhete Marine Station (CCMAR, University of Algarve, Faro, Portugal). Fish were maintained for 60 days in 1000 L tanks with running seawater (36 ppt) at a density 9–10 kg m^–3^ and fed 2% ration (fish wet weight, Sorgal, S.A., Portugal: Balance 3) once daily until the start of the experiment (all food was consumed by the fish). The experiment was setup by transferring the fish into 100 L flow-through tanks (without re-circulation) at a density of four fish (250 g body weight) per tank, in a total of six tanks. Temperature and photoperiod were natural (September-October, Algarve, Portugal) and the feeding regime was maintained as above during the 30 days of the experiment. No mortality was observed during the experiments. Fish were weighted and measured at the beginning and at the end of the experiment.

Each treatment (control and high *p*CO_2_, herein named 400 μatm CO_2_ and 1200 μatm CO_2_, respectively) had three replicates (3 × 100 L flow-through tank with four fish per tank). To achieve elevated levels of CO_2_, the rate of injection in the water was controlled by the pH level of seawater using pH probes connected to CO_2_ injection controllers (EXAxt PH450G; Yokogawa Iberia-Portugal). Each independent header-tank was gassed with CO_2_, thus obtaining two groups constantly maintained at 400 (control, no CO_2_ injection), and 1200 (high) μatm CO_2_. Seawater pH (NBS scale) was measured daily to calibrate the automated negative feedback system for CO_2_ injection (details in Gregório et al., 2019). Total alkalinity (TA) was measured using a combination DL15 titrator and a DG115-SC probe (Mettler-Toledo) using certified acid titrant (0.1 M HCl, Fluka Analytical, Sigma-Aldrich). Based on the measurements of salinity, temperature, alkalinity and pH, water *p*CO_2_ was calculated using CO2Calc Software (version 1.0.4; Robbins et al., 2010).

After 30 days of the experiment onset, fish were fasted for 24 h, and sampled between 9:30 and 11:00 AM under anesthesia in 2-phenoxyethanol (1: 10,000 v/v; Sigma–Aldrich, St. Louis, MO, United States). Fish were sacrificed by decapitation. The whole intestine was isolated and clamped (from pyloric caeca to anal sphincter) with two hemostatic forceps. Next the intestinal fluid was collected into a sterile vial for DNA extraction. The whole procedure was done in a flow cabinet, on an ice tray covered with a fresh disposable sterile cloth and using fresh disposable sterile dissecting tools for each fish.

### DNA Extraction

DNA extraction was conducted in fresh intestinal fluid samples, as soon as the animals were dissected, using the EZNA stool DNA kit (Omega Bio-tek, United States; 50 preps) following the manufacturer instructions in the Pathogen Detection Protocol. After extraction, DNA was stored at −20°C until used.

### PCR, Cloning, and Sequencing

All DNA samples obtained from the 24 animals (4 fish per tank and three tanks per treatment) were subjected to PCR, targeting the 16S rRNA coding gene. For that the primer pair 27F and 533R (Piazzon et al., 2017) was used, allowing the amplification of a 507 bp fragment containing the hypervariable regions V1–V3. PCR reactions were prepared in a PCR workstation to a final volume of 50 μl, containing: 5 μl of MTP Taq 10× Buffer, 2 mM of MgCl_2_, 200 μM of each dNTP, 200 nM of each primer, 2.5 U of MTP Taq DNA polymerase (Sigma-Aldrich Inc., United States) and 5 μl of DNA. Dedicated micropipettes and filtered tips were used throughout. Amplification reactions were done in a T100 Thermal Cycler (Bio-Rad Laboratories, Inc.) with the following program: initial 5 min at 95°C, followed by 30 cycles of 30 s at 94°C, 30 s at 53°C, and 30 s at 72°C, and a final extension of 5 min at 72°C. The round of PCR reactions was prepared to include three negative controls and a positive control (*Escherichia coli* DNA). Ten microliters of each PCR product were visualized under UV light after agarose gel electrophoresis (1.5% agarose in TAE 1x) and staining with ethidium bromide. All the negative controls showed no amplification band and the PCR products were processed further.

The amplicons obtained from each of the 4 fish from each tank were combined and ligated into the cloning vector using the pGEM-T Easy System I kit (Promega, United States) according to the manufacturer’s instructions. Each of these ligations, in a total of six ligations, was used to transform competent XL1 Blue *E. coli* cells. Transformations were plated onto LB-agar plates containing 50 μg.ml^–1^ ampicillin and to which surface 40 μL of X-Gal solution (20 mg/ml solution, Thermo Scientific) and 40 μL of IPTG solution (100 mM solution, Thermo Scientific) had been evenly spread. After overnight incubation, the white colonies were transferred to PCR tubes containing the following mix: 5 μl of Dream Taq 10x Buffer, 2 mM of MgCl_2_, 200 μM of each dNTP, 200 nM of each primer (primers M13F and M13R from the pGEM vector) and 1 U of dream Taq DNA polymerase (Thermo Fischer Scientific, Inc., United States). Amplification reactions, including a negative control, were done in a T100 Thermal Cycler (Bio-Rad Laboratories, Inc.) with the following program: initial 5 min at 95°C, followed by 30 cycles of 30s at 94°C, 30s at 60°C, and 30 s at 72°C, and a final extension of 5 min at 72°C. After agarose gel electrophoresis (1.5% agarose in TAE 1×) and staining with ethidium bromide, the PCR products were visualized under UV light.

PCR products corresponding to the expected size fragment were purified using the EZNA Cycle Pure kit (Omega Bio-tek, United States), and selected for commercial sequencing (CCMAR, Portugal; Sanger sequencing) using primers T7 and SP6 from the pGEM vector. Because some of the amplified fragments (less than 10% of the total clones screened per combined sample) were either longer or shorter than the expected size, these were also purified and sequenced for subsequent analysis.

### Sequence Data Analysis and Taxonomic Identification

The sequences obtained were visualized with the sequence editor BioEdit (Hall, 1999) and cut by the primers (27F and 533R). Each sequence obtained was individually subjected to a BLAST (Basic Local Alignment Search Tool) analysis (blastn), with the application BLASTN 2.8.0 available at NCBI^1^. Sequences resulting from amplicons longer or shorter than the expected size were also analyzed in the BLAST nucleotide database. It was verified they corresponded to low complexity sequences and were hence removed from subsequent analysis. The software Decipher version 2.11.3 (Wright et al., 2012) was used to identify possible chimeras, which were also removed from the datasets. At least 30 quality sequences were obtained per combined sample (F1–F4, F9–F12, F17–F20 for 400 μatm CO_2_ and F5–F8, F13–F16, F21–F24for 1200 μatm CO_2_).

Following this initial screening, FASTA files were generated with the sequences obtained for each combined sample, in a total of 6 files. Taxonomy assignment was done using the RDP classifier (Wang et al., 2007), with a confidence threshold of 80%. Each FASTA file was also submitted to a BLASTN analysis using the database for 16S ribosomal RNA sequences (Bacteria and Archaea) and excluding non-cultured organisms. The files obtained from RDP and from BLASTN were imported into MEGAN version 6.12.0 (Huson et al., 2016), to estimate the taxonomic composition of each sample microbiota. MEGAN is a software package used to calculate and explore the taxonomic content of metagenomic data, namely of data obtained through the sequencing of microbiomes. In MEGAN the assignment of reads to taxa based on the BLASTN files (100 matches per read) was computed using the naive LCA (Lowest Common Ancestor) algorithm (Huson et al., 2007), with a minimum threshold of 50 for the bit disjointScore of alignments or “hits,” and a maximum threshold of 0.01 for the *E*-value. No discrepancies were found between the two taxonomic assignments.

### Statistics

Using the pipeline implemented in MEGAN, rarefaction curves were first obtained for each of the six combined samples, by plotting the number of observed OTUs (OTU-operational taxonomic unit), or number of leaves in the taxonomy tree, against the number of sequences, or number of reads sampled from leaves. Subsequently, diversity indexes (Shannon–Weaver and Simpson reciprocal) were calculated for each combined sample, as a measure of alpha-diversity. The hit maps [number of reads allocated to each OTU (Family level)] were imported to PRIMER-e, version 7 software (Massey University, Albany, New Zealand) to explore the variance in microbial communities and test beta-diversity through multivariate analyses, using PERMANOVA + add-on (Clarke et al., 2014). All data were square root transformed and a Bray-Curtis dissimilarity coefficient was used to construct a resemblance matrix, with data visualized using non-metric multidimensional scaling analysis (nMDS). SIMPROF analyses were also conducted to further test for structure in the data. SIMPER (Similarity percentages) was used to identify significant alteration in the composition of OTUs and which OTUs were mainly responding to the effect of elevated CO_2_.

## Results

Experimental conditions and seawater physicochemical parameters are given in Table 1, as mean values (with standard deviations) of the three tanks per CO_2_ concentration used.

**TABLE 1:** Experimental conditions and seawater physicochemical parameters.

	**400 μatm CO_2_**	**1200 μatm CO_2_**
pH (NBS)	8.14±0.02	7.75±0.02
pCO_2_ (μatm)	411±11	1130±55
Alkalinity (μMol kg^–1^ SW)	2526±29	2511±33
Salinity (ppt)	34.8 ± 0.1	
T (°C)	24.7 ± 0.07	

The effect of exposure to elevated *p*CO_2_ during the 30 days of the experiment did not significantly affect fish weight nor fish length (Supplementary Figure S1).

**FIGURE 1:**
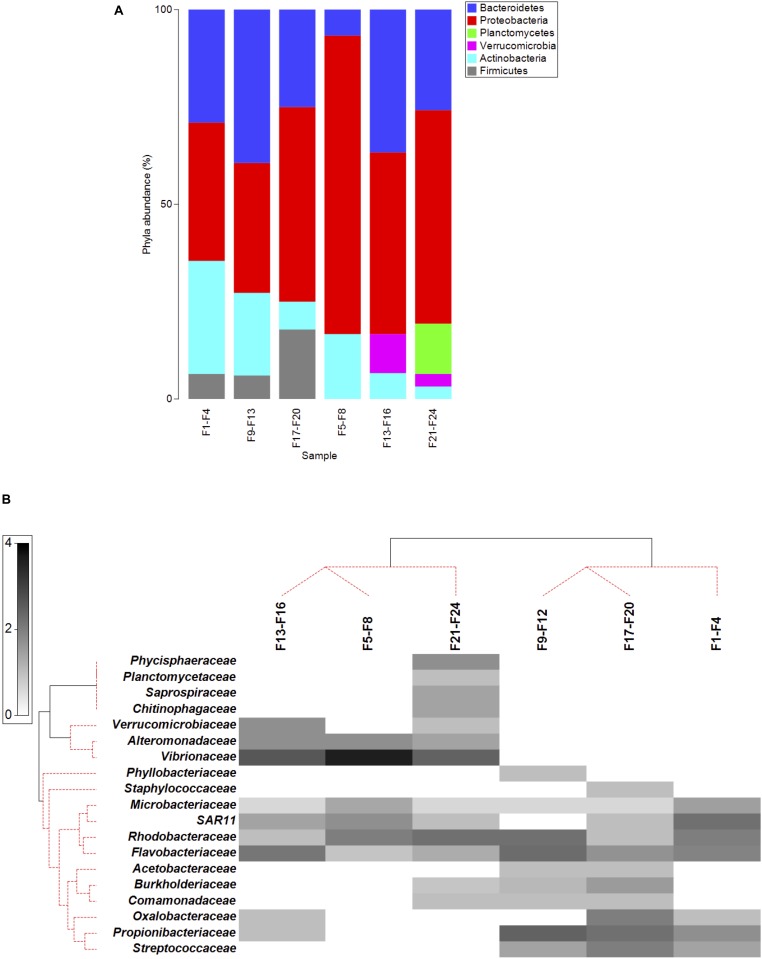
**(A)** Phyla abundance (%) in the microbiota of the intestinal fluid of *Sparus aurata* at 400 μatm CO_2_ (F1–F4, F9–F12, F17–F20) and at 1200 μatm CO_2_ (F5–F8, F13–F16, F21–F24). **(B)** Shade plot of abundance of the 19 OTUs (in the rows; Family level) identified in the six samples (in the columns) of *S. aurata* intestinal fluid. White space denotes absence of that OUT in that sample. Depth of gray scale is linearly proportional to a square-root transformation of abundance. The same transform was used for sample clustering and for OUT clustering. Samples were grouped using Bray–Curtis similarities on the transformed data, by hierarchical, unconstrained divisive clustering (UNCTREE), together with Type I SIMPROF tests. Two separated groups of samples (continuous lines), corresponding to the two CO_2_ levels under study, were supported by the SIMPROF test (significance level 5%). OTUs were also clustered, with the standard agglomerative method (group average linked), based on index of association resemblances computed on the transformed data, together with level Type 3 SIMPROF tests. Three coherent groups (continuous lines; cophenetic correlation: 0.86) were supported by the SIMPROF test (significance level 5%).

The data presented here are based on the assigned sequences (reads) obtained for each sample (Table 2). The classification of the sequences resulted in 19 OTUs assignments (family level) from 6 phyla (Figure 1A, Supplementary Figure S2, and Supplementary Files S1–S6). Based on the rarefaction curves (Supplementary Figure S2) and the Chao1 index (Table 2), satisfactory coverage of the existing bacterial OTUs was reached for the majority of the samples.

**TABLE 2:** Alpha diversity estimates through the determination of diversity indexes of the microbiota of the intestinal fluid of *Sparus aurata.*

**CO_2_ level (μatm CO_2_)**	**Sample**	**Shannon–Weaver**	**Simpson reciprocal**	**Chao1**	**Number of reads**	**OTUs (Family level)**	**Accession numbers**
400	F1–F4	2.643	5.727	7	32	7	MN240895–MN240926
	F9–F12	2.844	6.080	10	33	9	MN240969–MN241001
	F17–F20	2.980	6.323	14	32	11	MN240937–MN240968
1200	F5–F8	2.266	3.879	6	30	6	MN243925–MN243954
	F13–F16	2.777	5.631	10	30	9	MN241002–MN241031
	F21–F24	3.378	8.654	14	31	13	MN257910–MN257940

Proteobacteria, Bacteroidetes, and Actinobacteria, were the dominant phyla across the intestinal fluid microbial communities of the six samples, averaging 40, 31, and 19%, respectively, at 400 μatm CO_2_, and 60, 23, and 9%, respectively, at 1200 μatm CO_2_ (Figure 1A).

Four families from class Alpha-proteobacteria were detected in this work, *Acetobacteraceae*, *Phyllobacteriaceae*, *SAR11* and *Rhodobacteraceae*, although only the last was found in all of the six samples.

The Beta-proteobacteria, *Burkholderiaceae*, *Comamon- adaceae*, and *Oxalobacteraceae*, were detected in both treatments but not in all samples (Figure 1B).

The Gammaproteobacteria, *Vibrionaceae* and *Alteromon- adaceae*, were only present at elevated *p*CO_2_.

From phyla Bacteroidetes, the families *Saprospiraceae* and *Chitinophagaceae* were only found in one of the samples at elevated pCO_2_, whereas *Flavobacteriaceae* was found in all samples of both treatments.

Two families from phylum Actinobacteria were detected, *Propionibacteriacea* and *Microbacteriaceae*, with the last present in all samples.

Phylum Firmicutes was not detected at elevated pCO_2_, neither were phyla Planctomycetes and Verrucomicrobia, at 400 μatm CO_2_, although these two phyla were only detected in one of the high pCO_2_ samples.

The Shannon and Simpson diversity indexes (Table 2), as a measure of alpha diversity, were not significantly affected (*t*-test of the averaged values of the three samples per treatment) by elevated pCO_2_. However, microbial community compositions of the intestinal fluid at the two CO_2_ concentrations were found significantly different from each other (Bray-Curtis resemblance matrix, Monte–Carlo test *P* = 0.022, Supplementary Table S1), with no significant difference in dispersion of data within samples from the same treatment (PERMDISP, *p* > 0.05, Supplementary Table S1). Note that with a simple experimental design such as the present, the number of possible permutations is not sufficient for a proper PERMANOVA analysis. Instead the Monte Carlo test should be used (Clarke et al., 2014), as was done here. The differences between the two groups of samples were also supported by the LINKTREE (linkage tree) based on the Bray-Curtis similarity matrix, with samples clustering according to treatment (Figure 1B) (SIMPROF Type 1 test, *p* < 5%). Figure 1B also shows the OTUs clustering, with the standard agglomerative method (group average linked), based on the index of association resemblances. Three coherent groups (continuous lines) were supported by the SIMPROF Type 3 test (*p* < 5%; cophenetic correlation: 0.86), with two of the groups containing the OTUs only found at elevated *p*CO_2_.

**FIGURE 2:**
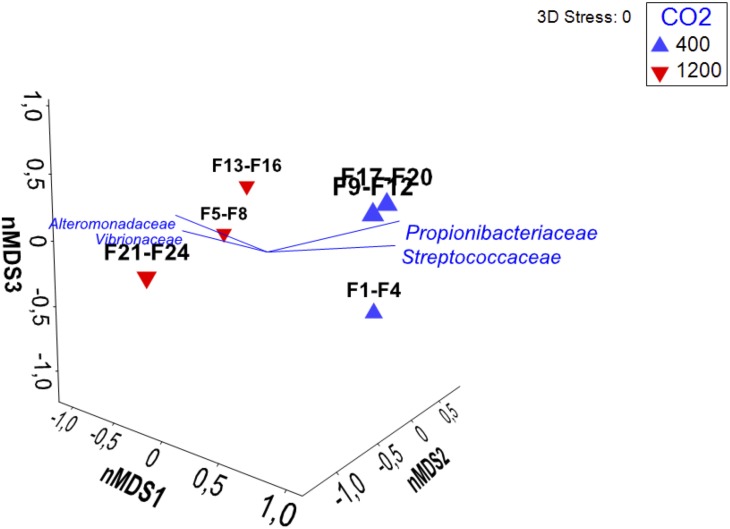
Non-metric multidimensional scaling (nMDS) plot based on Bray–Curtis similarity among microbial communities of *S. aurata* intestinal fluid samples, obtained from fish grown at 400 μatm CO_2_ (F1–4; F9–12; F17–20) and at 1200 μatm CO_2_ (F5–F8; F13–F16; F21–F24). The plot shows a clear change in community structure with the increase in pCO_2_ (*p* = 0.022, Monte–Carlo test). The vectors displayed indicate those OTUs identified as contributing most strongly toward microbiota similarity of samples from the same experimental group using SIMPER analysis. The vector direction for each OUT reflects the Pearson correlations of their square-root transformed counts with the two ordination axes, and length gives the multiple correlation coefficient from this linear regression on the ordination points.

The results of SIMPER (similarity percentages – species contributions, One-Way analysis) are shown in Supplementary Table S2. The average dissimilarity between samples from the two treatments was 60.97% whereas the similarity between samples from the same treatment was 65.24 and 60.93% for 400 μatm CO_2_ and 1200 μatm CO_2_, respectively. This analysis also revealed that the main OTUs driving the difference between the two treatments were *Vibrionaceae* (17.84%), *Propionibacteriaceae* (10.86%), *Alteromonadaceae* (9.94%), and *Streptococcaceae* (9.69%). This information was overlaid onto the result of the nMDS analysis (Figure 2), showing the significant difference in microbial community composition of the intestinal fluid between the two CO_2_ treatments.

## Discussion

This study was conducted as a preliminary approach to verify the putative effect of elevated *p*CO_2_ on the microbiota of the intestinal fluid.

For 30 days fish were exposed to control (400 μatm) and elevated (1200 μatm) CO_2_, with four fish in 100 L flow-through tanks (circa 1 kg fish/tank), allowing for complete water volume renovation every hour. Each treatment had three tanks and its own water circuit and fish were fed the same diet. Thus, the only difference between treatments was the injection of CO_2_ in the 1200 μatm tanks. Since the water microbial communities between the control and elevated pCO_2_ tanks were not expected to be altered with this experimental setup, we did not analyse water samples in our work.

The OTUs detected in the fish intestinal fluid belonged to 6 phyla, commonly found in marine fish gut (Sullam et al., 2012; Givens et al., 2015; Merrifield and Rodiles, 2015; Tran et al., 2017; Wang et al., 2018).

The observation that Proteobacteria, Bacteroidetes Actinobacteria and Firmicutes were the phyla detected in the control CO_2_ concentration (400 μatm CO_2_) is consistent with previously published studies on seabream GIT microbiota, either including the luminal and the mucosa-associated microbiota (Estruch et al., 2015), only the mucosa (Kormas et al., 2014) or only the luminal (Nikouli et al., 2018).

The remaining two phyla, Planctomycetes and Verrucomicrobia, were only detected at elevated pCO_2_ although not in all of the samples. These results showed some variation in intestinal fluid microbiota between replicate tanks, mainly at elevated pCO_2_, but no significant difference in dispersion of data was found (PERMDISP, *p* > 0.05, Supplementary Table S1). Both phyla are known to sporadically occur as subdominant phyla in microbiota (Ikeda-Ohtsubo et al., 2018) but their contribution to the structure and function of fish microbiota is not further explored in the literature.

Despite the differences in bacterial communities between the two treatments, three families from three different phyla were present in all of the samples: *Flavobacteriaceae* (Bacteroidetes), *Rhodobacteraceae* (Alphaproteobacteria), and *Microbacteriaceae* (Actinobacteria). These families were also identified in seabream by other authors (Estruch et al., 2015; Nikouli et al., 2018). A closer examination at the genus level (Supplementary Files S1–S6) shows a great dispersion in the genera present even in samples from the same treatment, with most of the genera identified with a low CT. In microbiota studies, OTUs present across a range of samples being compared are usually considered as the core biome (Jones et al., 2018), so we propose those three families might constitute the core microbiota of seabream in our experimental conditions.

The consistent differences in intestinal fluid microbial communities between the two CO_2_ treatments were that phylum Firmicutes was not found in the bacterial communities of fish exposed to 1200 μatm CO_2_, whereas Proteobacteria relative abundance was increased at elevated pCO_2_, due to the presence of Gammaproteobacteria (*Vibrionaceae* and *Alteromonadaceae*), a class not present in the control samples. Similarly, an increase in *Vibrionaceae* and *Alteromonadaceae* at elevated pCO_2_ was also reported in tissues, skeleton, and mucus of colonies of the coral *Acropora eurystoma*, exposed to pH 7.3 for 10 weeks, compared with those exposed to ambient pH 8.2 (Meron et al., 2011). An increase in antibacterial activity was also seen and was attributed mostly to an increase in *Vibrio* sp., suggesting a mechanism for host invasion (Meron et al., 2011).

*Vibrionaceae*, *Alteromonadaceae*, and different genera of lactic acid bacteria in phylum Firmicutes have been described in marine fish (Hovda et al., 2007; Denev et al., 2009; Pérez et al., 2010; Roeselers et al., 2011). In the study by Piazzon et al. (2017) on the intestinal mucus of seabream, in fish farm rearing conditions, *Vibrionaceae* (*Photobacterium* and *Vibrio*) was the dominant family in all diet groups tested. However, in a previous study by Estruch et al. (2015), using closed recirculation systems, *Vibrionaceae* was found in the stomach but not in the foregut, midgut or hindgut of seabream. In another study with seabream (Nikouli et al., 2018), from different commercial aquaculture farms in Greece, only Pseudomonadales, from the Gammaproteobacteria, were present.

These three studies differ in the allochthonous vs. autochthonous proportion of microbiota that was sampled, in experimental setup (water circulation, diet, feeding time, duration of the experiment) and in the environmental conditions (salinity, temperature). Having in view how sensitive the GIT microbiota is to any of those variables (Wang et al., 2018), these differences are most probably at the root of the apparently conflicting results and show that comparison between microbiota data sets needs to be made with caution and studies need to report the details of the sample preparation.

In our work, the *Vibrionaceae* present were attributed to genera *Vibrio* and *Lucibacterium* (*Vibrio harveyi*, Farmer et al., 2005), albeit with a confidence threshold (CT) frequently lower that 80% (Supplementary Files S4–S6). *Photobacterium* was not found in our samples.

*Vibrio* is one of the most important bacterial genera in aquaculture, with both pathogenic and probiotic species (Vandenberghe et al., 2003). *Vibrio* species are motile and facultative anaerobes. Although some *Vibrio* are clearly identified pathogens of marine organisms, as is *Vibrio harveyi*, it has been postulated that many *Vibrio* species could be not true pathogens, but rather opportunistic pathogens, whose virulence is accentuated under intensive aquaculture conditions (Thompson et al., 2004). Many *Vibrio* species are known to produce hydrolytic enzymes (amylase, lipase, chitinase) and in this way they can act as symbionts assisting in the breakdown of dietary components (Ray et al., 2012). However, whether or not the *Vibrio* detected at elevated *p*CO_2_ can behave as pathogens clearly needs further attention as this could add to the already described impact of elevated pCO_2_ on the immune system of fish (Kaya et al., 2013; Bresolin de Souza et al., 2014).

All the *Alteromonadaceae* present in our samples were attributed to genus *Aliiglaciecola* (CT 78%). *Alteromonadaceae* are aerobic and motile and related to polysaccharide degradation. It was observed that *SAR11* abundance is increased in the presence of *Alteromonadaceae* (Wietz et al., 2015) an effect attributed to cross-feeding on alginate monomers and other metabolic products released by alginolytic *Alteromonadaceae* (Wietz et al., 2015). In our study *SAR11* (CT 100%, *Candidatus pelagibacter*, Supplementary Files S1–S6) was consistently detected in all of the samples from elevated *p*CO_2_, but in only two of the control samples. *SAR11* are chemoheterotrophic marine bacteria with a large impact on the cycling of carbon and other important nutrients in the oceans, capable of withstanding and adjust quickly to fluctuations in the availability of nutrients (Sowell et al., 2008).

The presence of Gammaproteobacteria at elevated *p*CO_2_ was coincident with the absence of Firmicutes. Firmicutes and Bacteroidetes are known to contribute to carbohydrates and/or proteins fermentation in the intestine to help the host acquire nutrients from the diet (Spor et al., 2011). In our study phylum Bacteroidetes was present at 1200 μatm CO_2_, but phylum Firmicutes could not be detected in this treatment. However, family *Streptococcaceae*, genus *Streptoccocus* (CT 100%, Supplementary Files 1–3) was detected in all control samples. *Streptococcus* was also the most abundant Firmicutes genus found in seabream by Estruch et al. (2015). Lactic acid bacteria (LAB) such as *Streptococcus*, and Firmicutes in general, are thought to be important to host carbohydrate metabolism through fermentation and have been associated with efficient dietary energy harvest (Turnbaugh et al., 2006; Guo et al., 2008; Krajmalnik-Brown et al., 2012). Intestinal composition of LAB is known to vary with season in different species (Wang et al., 2018, and references therein) and also with the feeding regime (Parris et al., 2019). LAB are among the microbiota bacteria most responsive to feeding, with many taxa showing a positive response, a factor that may also contribute to their role in energy extraction from food. Interestingly, *Propionibacteriaceae*, were also negatively affected by elevated *p*CO_2_. Although present in all of the control tanks, this family was found in only one of the elevated pCO_2_ samples. *Propionibacterium*, the genus detected (CT 100%, Supplementary Files S1–S3, S5), is a lipophilic facultative anaerobe that feeds on lactate, and is used in calves as a probiotic to counteract low ruminal pH that might cause subacute ruminal acidosis or SARA (Brugman et al., 2018).

## Conclusion

Our observations provide a first glimpse of how the bacterial community in fish gut can be affected by exposure to elevated *p*CO_2_. The data suggests that the chemical and physiological compensatory processes, taking place in the intestinal lumen of the fish, provoke the significant dysbiosis seen at elevated pCO_2_, by affecting mainly the LAB in the intestinal fluid bacterial communities, while favoring bacteria with other pathways of polysaccharide and protein degradation. This shift might be related to the delayed digestion observed in fish under hypercapnia. Since the bacterial groups replacing LAB can harbor putative pathogens, future studies characterizing the immune response of fishes to elevated pCO_2_ will need to analyze and take into account alterations in fish microbiota.

## Data Availability Statement

The datasets generated for this study can be found in the NCBI Nucleotide Database F1–F4: MN240895–MN240926, F9–F12: MN240969–MN241001, F17–F20: MN240937–MN240968, F5–F8: MN243925–MN243954, F13–F16: MN241002–MN241031, and F21–F24: MN257910–MN257940.

## Author Contributions

FF and JF planned the work and designed the study. JF sampled the animals. FF and RC performed the laboratory work and the data analyses. FF, RC, and JF wrote the manuscript and read and approved the content of the manuscript.

## Conflict of Interest

The authors declare that the research was conducted in the absence of any commercial or financial relationships that could be construed as a potential conflict of interest.

## Footnotes

1 https://eee.ncbi.nim.nih.gov
