# Supplementary material for: Impact of Ocean Acidification on the Intestinal Microbiota of the Marine Sea Bream (Sparus aurata L.)
Source: Front Physiol. 2019 Nov 28;10:1446. doi: 10.3389/fphys.2019.01446 (PMC6893888; doi:10.3389/fphys.2019.01446)
Supplement: Supplementary file 3 [file Data_Sheet_1.PDF]

Classifier: RDP Naive Bayesian rRNA Classifier Version 2.11, September 2015
Taxonomical Hierarchy: RDP 16S rRNA training set 16
Query File: F1-F4 GenBank and RDP.fas
Submit Date: Wed Jul 10 06:49:28 EDT 2019
Confidence threshold (for classification to Root ONLY): 95%
Symbol +/- indicates predicted sequence orientation

F1I.20_;+;Root;100%;Bacteria;100%;"Bacteroidetes";
100%;Flavobacteriia;98%;"Flavobacteriales";98%;Flavobacteriaceae;
77%;Kordia;13%
F1I.24_;+;Root;100%;Bacteria;100%;"Bacteroidetes";
100%;Flavobacteriia;98%;"Flavobacteriales";98%;Flavobacteriaceae;
77%;Kordia;14%
F1I30;+;Root;100%;Bacteria;100%;"Bacteroidetes";100%;Flavobacteriia;
98%;"Flavobacteriales";98%;Flavobacteriaceae;77%;Kordia;13%
F1II.30_;+;Root;100%;Bacteria;100%;"Bacteroidetes";
100%;Flavobacteriia;99%;"Flavobacteriales";99%;Flavobacteriaceae;
80%;Wenyingzhuangia;15%
F1I.21_;+;Root;100%;Bacteria;100%;"Proteobacteria";
100%;Betaproteobacteria;100%;Burkholderiales;100%;Oxalobacteraceae;
100%;Herbaspirillum;100%
F1II.8_;+;Root;100%;Bacteria;100%;"Proteobacteria";
100%;Betaproteobacteria;100%;Burkholderiales;100%;Oxalobacteraceae;
100%;Herbaspirillum;100%
F1I.25_;+;Root;100%;Bacteria;100%;"Proteobacteria";
100%;Alphaproteobacteria;100%;Rhodobacterales;100%;Rhodobacteraceae;
100%;Roseovarius;56%
F1II.25_;+;Root;100%;Bacteria;100%;"Actinobacteria";
100%;Actinobacteria;100%;Actinobacteridae;100%;Actinomycetales;
100%;Propionibacterineae;100%;Propionibacteriaceae;
100%;Propionibacterium;100%
F1II.2_;+;Root;100%;Bacteria;100%;"Actinobacteria";
100%;Actinobacteria;100%;Actinobacteridae;100%;Actinomycetales;
100%;Propionibacterineae;100%;Propionibacteriaceae;
100%;Propionibacterium;100%
F1II.15;+;Root;100%;Bacteria;100%;"Actinobacteria";
100%;Actinobacteria;100%;Actinobacteridae;100%;Actinomycetales;
100%;Propionibacterineae;100%;Propionibacteriaceae;
100%;Propionibacterium;100%
F1II.3_;+;Root;100%;Bacteria;100%;"Proteobacteria";
100%;Alphaproteobacteria;100%;SAR11;100%;Candidatus Pelagibacter;
100%
F1II.23;+;Root;100%;Bacteria;100%;"Proteobacteria";
100%;Alphaproteobacteria;100%;SAR11;100%;Candidatus Pelagibacter;
100%
F1II.26;+;Root;100%;Bacteria;100%;"Proteobacteria";
100%;Alphaproteobacteria;100%;SAR11;100%;Candidatus Pelagibacter;
100%
F1II.4;+;Root;100%;Bacteria;100%;"Actinobacteria";
100%;Actinobacteria;100%;Actinobacteridae;100%;Actinomycetales;
100%;Micrococcineae;100%;Microbacteriaceae;100%;Microcella;51%
F1II.1;+;Root;100%;Bacteria;100%;"Actinobacteria";
100%;Actinobacteria;100%;Actinobacteridae;100%;Actinomycetales;

100%;Micrococcineae;100%;Microbacteriaceae;100%;Rhodoluna;19%
F1II.28_;+;Root;100%;Bacteria;100%;"Actinobacteria";
100%;Actinobacteria;100%;Actinobacteridae;100%;Actinomycetales;
100%;Micrococcineae;100%;Microbacteriaceae;100%;Pontimonas;93%
F1II.29;+;Root;100%;Bacteria;100%;"Actinobacteria";
100%;Actinobacteria;100%;Actinobacteridae;100%;Actinomycetales;
100%;Micrococcineae;100%;Microbacteriaceae;100%;Agrococcus;53%
F1II.32_;+;Root;100%;Bacteria;100%;"Actinobacteria";
100%;Actinobacteria;100%;Actinobacteridae;100%;Actinomycetales;
100%;Micrococcineae;100%;Microbacteriaceae;100%;Rhodoluna;49%
F1I.27;+;Root;100%;Bacteria;100%;"Bacteroidetes";
100%;Flavobacteriia;100%;"Flavobacteriales";100%;Flavobacteriaceae;
100%;Flavivirga;28%
F1II.17_;+;Root;100%;Bacteria;100%;"Proteobacteria";
100%;Alphaproteobacteria;100%;SAR11;100%;Candidatus Pelagibacter;
100%
F1II.9_;+;Root;100%;Bacteria;100%;"Proteobacteria";
100%;Alphaproteobacteria;100%;SAR11;100%;Candidatus Pelagibacter;
100%
F1II.31_;+;Root;100%;Bacteria;100%;"Proteobacteria";
100%;Alphaproteobacteria;100%;Rhodobacterales;100%;Rhodobacteraceae;
100%;Roseicyclus;46%
F1II.35_;+;Root;100%;Bacteria;100%;"Proteobacteria";
100%;Alphaproteobacteria;100%;Rhodobacterales;100%;Rhodobacteraceae;
100%;Litoreibacter;100%
F1II.21_;+;Root;100%;Bacteria;100%;"Proteobacteria";
100%;Alphaproteobacteria;100%;Rhodobacterales;80%;Rhodobacteraceae;
80%;Hasllibacter;27%
F1II.27_;+;Root;100%;Bacteria;100%;Firmicutes;100%;Bacilli;
100%;Lactobacillales;100%;Streptococcaceae;100%;Streptococcus;100%
F1II.24;+;Root;100%;Bacteria;100%;Firmicutes;100%;Bacilli;
100%;Lactobacillales;100%;Streptococcaceae;100%;Streptococcus;100%
F1II.14;+;Root;100%;Bacteria;100%;"Bacteroidetes";
100%;Flavobacteriia;99%;"Flavobacteriales";99%;Flavobacteriaceae;
80%;Kordia;22%
F1II.6;+;Root;100%;Bacteria;100%;"Bacteroidetes";
100%;Flavobacteriia;99%;"Flavobacteriales";99%;Flavobacteriaceae;
80%;Kordia;22%
F1I.23;+;Root;100%;Bacteria;100%;"Bacteroidetes";
100%;Flavobacteriia;99%;"Flavobacteriales";99%;Flavobacteriaceae;
80%;Kordia;22%
F1II.5;+;Root;100%;Bacteria;100%;"Actinobacteria";
100%;Actinobacteria;100%;Actinobacteridae;100%;Actinomycetales;
100%;Micrococcineae;100%;Microbacteriaceae;100%;Pontimonas;93%
F1II.13_;+;Root;100%;Bacteria;100%;"Actinobacteria";
100%;Actinobacteria;100%;Actinobacteridae;100%;Actinomycetales;
100%;Micrococcineae;100%;Microbacteriaceae;100%;Pontimonas;93%
F1I.26;+;Root;100%;Bacteria;100%;"Bacteroidetes";
100%;Flavobacteriia;100%;"Flavobacteriales";100%;Flavobacteriaceae;
100%;Flavivirga;28%
