# Supplementary material for: Impact of Ocean Acidification on the Intestinal Microbiota of the Marine Sea Bream (Sparus aurata L.)
Source: Front Physiol. 2019 Nov 28;10:1446. doi: 10.3389/fphys.2019.01446 (PMC6893888; doi:10.3389/fphys.2019.01446)
Supplement: Supplementary file 4 [file Data_Sheet_2.PDF]

Classifier: RDP Naive Bayesian rRNA Classifier Version 2.11, September 2015
Taxonomical Hierarchy: RDP 16S rRNA training set 16
Query File: F9-F12 GenBank.fas
Submit Date: Wed Jul 10 07:20:23 EDT 2019
Confidence threshold (for classification to Root ONLY): 95%
Symbol +/- indicates predicted sequence orientation

F9.36;+;Root;100%;Bacteria;100%;"Bacteroidetes";100%;Flavobacteriia;98%;"Flavobacteriales";98%;Flavobacteriaceae;77%;Kordia;13%
F9.37;+;Root;100%;Bacteria;100%;"Bacteroidetes";100%;Flavobacteriia;98%;"Flavobacteriales";98%;Flavobacteriaceae;77%;Kordia;14%
F9.39;+;Root;100%;Bacteria;100%;"Bacteroidetes";100%;Flavobacteriia;98%;"Flavobacteriales";98%;Flavobacteriaceae;77%;Kordia;13%
F9.42;+;Root;100%;Bacteria;100%;"Bacteroidetes";100%;Flavobacteriia;98%;"Flavobacteriales";98%;Flavobacteriaceae;77%;Kordia;14%
F9.43;+;Root;100%;Bacteria;100%;"Bacteroidetes";100%;Flavobacteriia;98%;"Flavobacteriales";98%;Flavobacteriaceae;77%;Kordia;14%
F9.44;+;Root;100%;Bacteria;100%;"Bacteroidetes";100%;Flavobacteriia;98%;"Flavobacteriales";98%;Flavobacteriaceae;77%;Kordia;13%
F9.45;+;Root;100%;Bacteria;100%;"Bacteroidetes";100%;Flavobacteriia;98%;"Flavobacteriales";98%;Flavobacteriaceae;77%;Kordia;14%
F9.46;+;Root;100%;Bacteria;100%;"Bacteroidetes";100%;Flavobacteriia;98%;"Flavobacteriales";98%;Flavobacteriaceae;77%;Kordia;13%
F9.48;+;Root;100%;Bacteria;100%;"Bacteroidetes";100%;Flavobacteriia;98%;"Flavobacteriales";98%;Flavobacteriaceae;77%;Kordia;14%
F9.49;+;Root;100%;Bacteria;100%;"Bacteroidetes";100%;Flavobacteriia;98%;"Flavobacteriales";98%;Flavobacteriaceae;77%;Kordia;14%
F9.7;+;Root;100%;Bacteria;100%;"Proteobacteria";100%;Betaproteobacteria;100%;Burkholderiales;100%;Comamonadaceae;100%;Variovorax;100%
F9.35;+;Root;100%;Bacteria;100%;Firmicutes;100%;Bacilli;100%;Lactobacillales;100%;Streptococcaceae;100%;Streptococcus;100%
F9.2;+;Root;100%;Bacteria;100%;Firmicutes;100%;Bacilli;100%;Lactobacillales;100%;Streptococcaceae;100%;Streptococcus;100%
F9.40;+;Root;100%;Bacteria;100%;"Proteobacteria";100%;Alphaproteobacteria;100%;Rhodobacterales;100%;Rhodobacteraceae;100%;Oceanicola;72%
F9.47;+;Root;100%;Bacteria;100%;"Proteobacteria";100%;Alphaproteobacteria;100%;Rhodobacterales;100%;Rhodobacteraceae;100%;Oceanicola;72%
F9.41;+;Root;100%;Bacteria;100%;"Proteobacteria";100%;Alphaproteobacteria;100%;Rhodobacterales;100%;Rhodobacteraceae;100%;Leisingera;100%
F9.53;+;Root;100%;Bacteria;100%;"Bacteroidetes";100%;Flavobacteriia;100%;"Flavobacteriales";100%;Flavobacteriaceae;100%;Tenacibaculum;93%
F9.64;+;Root;100%;Bacteria;100%;"Bacteroidetes";100%;Flavobacteriia;100%;"Flavobacteriales";100%;Flavobacteriaceae;100%;Flavivirga;24%
F9.63;+;Root;100%;Bacteria;100%;"Bacteroidetes";100%;Flavobacteriia;100%;"Flavobacteriales";100%;Flavobacteriaceae;100%;Arenitalea;100%
F9.38;+;Root;100%;Bacteria;100%;"Proteobacteria";100%;Alphaproteobacteria;100%;Rhizobiales;100%;Phyllobacteriaceae;100%;Pseudahrensia;74%

F9.54;+;Root;100%;Bacteria;100%;"Actinobacteria";
100%;Actinobacteria;100%;Actinobacteridae;100%;Actinomycetales;
100%;Propionibacterineae;100%;Propionibacteriaceae;
100%;Propionibacterium;100%
F9.59;+;Root;100%;Bacteria;100%;"Actinobacteria";
100%;Actinobacteria;100%;Actinobacteridae;100%;Actinomycetales;
100%;Propionibacterineae;100%;Propionibacteriaceae;
100%;Propionibacterium;100%
F9.55;+;Root;100%;Bacteria;100%;"Actinobacteria";
100%;Actinobacteria;100%;Actinobacteridae;100%;Actinomycetales;
100%;Propionibacterineae;100%;Propionibacteriaceae;
100%;Propionibacterium;100%
F9.56;+;Root;100%;Bacteria;100%;"Actinobacteria";
100%;Actinobacteria;100%;Actinobacteridae;100%;Actinomycetales;
100%;Propionibacterineae;100%;Propionibacteriaceae;
100%;Propionibacterium;100%
F9.57;+;Root;100%;Bacteria;100%;"Actinobacteria";
100%;Actinobacteria;100%;Actinobacteridae;100%;Actinomycetales;
100%;Propionibacterineae;100%;Propionibacteriaceae;
100%;Propionibacterium;100%
F9.58;+;Root;100%;Bacteria;100%;"Actinobacteria";
100%;Actinobacteria;100%;Actinobacteridae;100%;Actinomycetales;
100%;Propionibacterineae;100%;Propionibacteriaceae;
100%;Propionibacterium;100%
F9.21;+;Root;100%;Bacteria;100%;"Actinobacteria";
100%;Actinobacteria;100%;Actinobacteridae;100%;Actinomycetales;
100%;Micrococcineae;100%;Microbacteriaceae;100%;Rhodoluna;19%
F9.20;+;Root;100%;Bacteria;100%;"Proteobacteria";
100%;Betaproteobacteria;100%;Burkholderiales;100%;Burkholderiaceae;
100%;Ralstonia;100%
F9.1;+;Root;100%;Bacteria;100%;"Proteobacteria";
100%;Betaproteobacteria;100%;Burkholderiales;100%;Burkholderiaceae;
100%;Burkholderia;100%
F9.3;+;Root;100%;Bacteria;100%;"Proteobacteria";
100%;Betaproteobacteria;100%;Burkholderiales;100%;Burkholderiaceae;
100%;Lautropia;100%
F9.52;+;Root;100%;Bacteria;100%;"Proteobacteria";
100%;Alphaproteobacteria;100%;Rhodobacterales;98%;Rhodobacteraceae;
98%;Ahrensia;97%
F9.25;+;Root;100%;Bacteria;100%;"Proteobacteria";
100%;Alphaproteobacteria;100%;Rhodobacterales;100%;Rhodobacteraceae;
100%;Oceanicola;72%
F9.26;+;Root;100%;Bacteria;100%;"Proteobacteria";
100%;Alphaproteobacteria;100%;Rhodospirillales;
100%;Acetobacteraceae;100%;Acidisphaera;68%
