# Supplementary material for: Impact of Ocean Acidification on the Intestinal Microbiota of the Marine Sea Bream (Sparus aurata L.)
Source: Front Physiol. 2019 Nov 28;10:1446. doi: 10.3389/fphys.2019.01446 (PMC6893888; doi:10.3389/fphys.2019.01446)
Supplement: Supplementary file 5 [file Data_Sheet_3.PDF]

Classifier: RDP Naive Bayesian rRNA Classifier Version 2.11, September 2015
Taxonomical Hierarchy: RDP 16S rRNA training set 16
Query File: F17-F20 GenBank.fas
Submit Date: Wed Jul 10 07:11:13 EDT 2019
Confidence threshold (for classification to Root ONLY): 95%
Symbol +/- indicates predicted sequence orientation

F17.1;+;Root;100%;Bacteria;100%;"Actinobacteria";100%;Actinobacteria;100%;Actinobacteridae;100%;Actinomycetales;100%;Propionibacterineae;100%;Propionibacteriaceae;100%;Propionibacterium;100%
F17.9_;+;Root;100%;Bacteria;100%;"Actinobacteria";100%;Actinobacteria;100%;Actinobacteridae;100%;Actinomycetales;100%;Propionibacterineae;100%;Propionibacteriaceae;100%;Propionibacterium;100%
F17.37_;+;Root;100%;Bacteria;100%;"Actinobacteria";100%;Actinobacteria;100%;Actinobacteridae;100%;Actinomycetales;100%;Propionibacterineae;100%;Propionibacteriaceae;100%;Propionibacterium;100%
F17.45_;+;Root;100%;Bacteria;100%;"Actinobacteria";100%;Actinobacteria;100%;Actinobacteridae;100%;Actinomycetales;100%;Propionibacterineae;100%;Propionibacteriaceae;100%;Propionibacterium;100%
F17.50_;+;Root;100%;Bacteria;100%;"Actinobacteria";100%;Actinobacteria;100%;Actinobacteridae;100%;Actinomycetales;100%;Propionibacterineae;100%;Propionibacteriaceae;100%;Propionibacterium;100%
F17.2_;+;Root;100%;Bacteria;100%;Firmicutes;100%;Bacilli;100%;Lactobacillales;100%;Streptococcaceae;100%;Streptococcus;100%
F17.14;+;Root;100%;Bacteria;100%;Firmicutes;100%;Bacilli;100%;Lactobacillales;100%;Streptococcaceae;100%;Streptococcus;100%
F17.41_;+;Root;100%;Bacteria;100%;Firmicutes;100%;Bacilli;100%;Lactobacillales;100%;Streptococcaceae;100%;Streptococcus;100%
F17.49_;+;Root;100%;Bacteria;100%;Firmicutes;100%;Bacilli;100%;Lactobacillales;100%;Streptococcaceae;100%;Streptococcus;100%
F17.18_;+;Root;100%;Bacteria;100%;"Proteobacteria";100%;Betaproteobacteria;100%;Burkholderiales;100%;Burkholderiaceae;100%;Ralstonia;100%
F17.36_;+;Root;100%;Bacteria;100%;"Proteobacteria";100%;Betaproteobacteria;100%;Burkholderiales;100%;Burkholderiaceae;100%;Ralstonia;100%
F17.8;+;Root;100%;Bacteria;100%;"Proteobacteria";100%;Betaproteobacteria;100%;Burkholderiales;100%;Burkholderiaceae;100%;Burkholderia;100%
F17.12_;+;Root;100%;Bacteria;100%;"Proteobacteria";100%;Betaproteobacteria;100%;Burkholderiales;100%;Burkholderiaceae;100%;Burkholderia;100%
F17.46;+;Root;100%;Bacteria;100%;"Proteobacteria";100%;Betaproteobacteria;100%;Burkholderiales;100%;Burkholderiaceae;100%;Lautropia;100%
F17.17;+;Root;100%;Bacteria;100%;"Proteobacteria";100%;Betaproteobacteria;100%;Burkholderiales;100%;Burkholderiaceae;100%;Lautropia;100%

F17.11_;+;Root;100%;Bacteria;100%;"Proteobacteria";
100%;Alphaproteobacteria;100%;Rhodobacterales;98%;Rhodobacteraceae;
98%;Ahrensia;97%
F17.38_;+;Root;100%;Bacteria;100%;"Proteobacteria";
100%;Betaproteobacteria;100%;Burkholderiales;100%;Oxalobacteraceae;
100%;Herbaspirillum;100%
F17.47;+;Root;100%;Bacteria;100%;"Proteobacteria";
100%;Betaproteobacteria;100%;Burkholderiales;100%;Oxalobacteraceae;
100%;Herbaspirillum;100%
F17.42_;+;Root;100%;Bacteria;100%;"Proteobacteria";
99%;Alphaproteobacteria;99%;SAR11;94%;Candidatus Pelagibacter;94%
F17.43;+;Root;100%;Bacteria;100%;"Proteobacteria";
100%;Betaproteobacteria;100%;Burkholderiales;100%;Comamonadaceae;
100%;Variovorax;100%
F17.51_;+;Root;100%;Bacteria;100%;Firmicutes;100%;Bacilli;
100%;Bacillales;100%;Staphylococcaceae;100%;Staphylococcus;100%
F17.3;+;Root;100%;Bacteria;100%;"Bacteroidetes";100%;Flavobacteriia;
98%;"Flavobacteriales";98%;Flavobacteriaceae;77%;Kordia;13%
F17.16;+;Root;100%;Bacteria;100%;"Bacteroidetes";
100%;Flavobacteriia;98%;"Flavobacteriales";98%;Flavobacteriaceae;
77%;Kordia;13%
F17.4;+;Root;100%;Bacteria;100%;"Bacteroidetes";100%;Flavobacteriia;
98%;"Flavobacteriales";98%;Flavobacteriaceae;77%;Kordia;14%
F17.5;+;Root;100%;Bacteria;100%;"Bacteroidetes";100%;Flavobacteriia;
98%;"Flavobacteriales";98%;Flavobacteriaceae;77%;Kordia;13%
F17.6;+;Root;100%;Bacteria;100%;"Bacteroidetes";100%;Flavobacteriia;
99%;"Flavobacteriales";99%;Flavobacteriaceae;80%;Kordia;22%
F17.10;+;Root;100%;Bacteria;100%;"Bacteroidetes";
100%;Flavobacteriia;99%;"Flavobacteriales";99%;Flavobacteriaceae;
80%;Wenyingzhuangia;15%
F17.13;+;Root;100%;Bacteria;100%;"Actinobacteria";
100%;Actinobacteria;100%;Actinobacteridae;100%;Actinomycetales;
100%;Micrococcineae;100%;Microbacteriaceae;100%;Pontimonas;93%
F17.40;+;Root;100%;Bacteria;100%;"Proteobacteria";
100%;Betaproteobacteria;100%;Burkholderiales;100%;Oxalobacteraceae;
100%;Herbaspirillum;100%
F17.44;+;Root;100%;Bacteria;100%;"Proteobacteria";
100%;Betaproteobacteria;100%;Burkholderiales;100%;Oxalobacteraceae;
100%;Herbaspirillum;100%
F17.7;+;Root;100%;Bacteria;100%;"Bacteroidetes";100%;Flavobacteriia;
99%;"Flavobacteriales";99%;Flavobacteriaceae;80%;Wenyingzhuangia;15%
F17.15;+;Root;100%;Bacteria;100%;"Proteobacteria";
100%;Alphaproteobacteria;100%;Rhodospirillales;
100%;Acetobacteraceae;100%;Acidisphaera;68%
