# Supplementary material for: Impact of Ocean Acidification on the Intestinal Microbiota of the Marine Sea Bream (Sparus aurata L.)
Source: Front Physiol. 2019 Nov 28;10:1446. doi: 10.3389/fphys.2019.01446 (PMC6893888; doi:10.3389/fphys.2019.01446)
Supplement: Supplementary file 6 [file Data_Sheet_4.PDF]

Classifier: RDP Naive Bayesian rRNA Classifier Version 2.11, September 2015
Taxonomical Hierarchy: RDP 16S rRNA training set 16
Query File: F5-F8 GenBank.fas
Submit Date: Wed Jul 10 07:31:13 EDT 2019
Confidence threshold (for classification to Root ONLY): 95%
Symbol +/- indicates predicted sequence orientation

F5.2_;+;Root;100%;Bacteria;100%;"Proteobacteria";100%;Gammaproteobacteria;100%;"Vibrionales";100%;Vibrionaceae;100%;Lucibacterium;66%
F5.4_;+;Root;100%;Bacteria;100%;"Proteobacteria";100%;Gammaproteobacteria;100%;"Vibrionales";100%;Vibrionaceae;100%;Vibrio;47%
F5.19_;+;Root;100%;Bacteria;100%;"Proteobacteria";100%;Gammaproteobacteria;100%;"Vibrionales";100%;Vibrionaceae;100%;Vibrio;57%
F5.20_;+;Root;100%;Bacteria;100%;"Proteobacteria";100%;Gammaproteobacteria;100%;"Vibrionales";100%;Vibrionaceae;100%;Vibrio;48%
F5.1;+;Root;100%;Bacteria;100%;"Proteobacteria";100%;Gammaproteobacteria;100%;"Vibrionales";100%;Vibrionaceae;100%;Lucibacterium;64%
F5.24;+;Root;100%;Bacteria;100%;"Proteobacteria";100%;Gammaproteobacteria;100%;"Vibrionales";100%;Vibrionaceae;100%;Vibrio;54%
F5.6_;+;Root;100%;Bacteria;100%;"Proteobacteria";100%;Gammaproteobacteria;100%;"Vibrionales";100%;Vibrionaceae;100%;Lucibacterium;52%
F5.11_;+;Root;100%;Bacteria;100%;"Proteobacteria";100%;Gammaproteobacteria;100%;"Vibrionales";100%;Vibrionaceae;100%;Vibrio;63%
F5.12_;+;Root;100%;Bacteria;100%;"Proteobacteria";100%;Gammaproteobacteria;100%;"Vibrionales";100%;Vibrionaceae;100%;Vibrio;48%
F5.15_;+;Root;100%;Bacteria;100%;"Proteobacteria";100%;Gammaproteobacteria;100%;"Vibrionales";100%;Vibrionaceae;100%;Vibrio;94%
F5.29_;+;Root;100%;Bacteria;100%;"Proteobacteria";100%;Gammaproteobacteria;100%;"Vibrionales";100%;Vibrionaceae;100%;Lucibacterium;64%
F5.31_;+;Root;100%;Bacteria;100%;"Proteobacteria";100%;Gammaproteobacteria;100%;"Vibrionales";100%;Vibrionaceae;100%;Lucibacterium;74%
F5.10_;+;Root;100%;Bacteria;100%;"Proteobacteria";100%;Gammaproteobacteria;100%;"Vibrionales";100%;Vibrionaceae;100%;Vibrio;63%
F5.16;+;Root;100%;Bacteria;100%;"Actinobacteria";100%;Actinobacteria;100%;Actinobacteridae;100%;Actinomycetales;100%;Micrococcineae;100%;Microbacteriaceae;100%;Rhodoluna;51%
F5.21;+;Root;100%;Bacteria;100%;"Actinobacteria";100%;Actinobacteria;100%;Actinobacteridae;100%;Actinomycetales;100%;Micrococcineae;100%;Microbacteriaceae;100%;Rhodoluna;49%
F5.28_;+;Root;100%;Bacteria;100%;"Actinobacteria";

100%;Actinobacteria;100%;Actinobacteridae;100%;Actinomycetales;
100%;Micrococcineae;100%;Microbacteriaceae;100%;Rhodoluna;58%
F5.9_;+;Root;100%;Bacteria;100%;"Actinobacteria";
100%;Actinobacteria;100%;Actinobacteridae;100%;Actinomycetales;
100%;Micrococcineae;100%;Microbacteriaceae;100%;Rhodoluna;67%
F5.30_;+;Root;100%;Bacteria;100%;"Actinobacteria";
100%;Actinobacteria;100%;Actinobacteridae;100%;Actinomycetales;
100%;Micrococcineae;100%;Microbacteriaceae;100%;Rhodoluna;49%
F5.3_;+;Root;100%;Bacteria;100%;"Proteobacteria";
100%;Alphaproteobacteria;100%;Rhodobacterales;100%;Rhodobacteraceae;
100%;Roseovarius;56%
F5.17_;+;Root;100%;Bacteria;100%;"Proteobacteria";
100%;Alphaproteobacteria;100%;Rhodobacterales;100%;Rhodobacteraceae;
100%;Roseovarius;54%
F5.25;+;Root;100%;Bacteria;100%;"Proteobacteria";
100%;Alphaproteobacteria;100%;Rhodobacterales;100%;Rhodobacteraceae;
100%;Roseovarius;57%
F5.14_;+;Root;100%;Bacteria;100%;"Proteobacteria";
100%;Alphaproteobacteria;100%;Rhodobacterales;100%;Rhodobacteraceae;
100%;Roseovarius;59%
F5.18;+;Root;100%;Bacteria;100%;"Proteobacteria";
100%;Alphaproteobacteria;100%;SAR11;100%;Candidatus Pelagibacter;
100%
F5.26_;+;Root;100%;Bacteria;100%;"Proteobacteria";
100%;Alphaproteobacteria;100%;SAR11;100%;Candidatus Pelagibacter;
100%
F5.22_;+;Root;100%;Bacteria;100%;"Proteobacteria";
100%;Alphaproteobacteria;100%;SAR11;100%;Candidatus Pelagibacter;
100%
F5.7;+;Root;100%;Bacteria;100%;"Proteobacteria";
100%;Gammaproteobacteria;100%;Alteromonadales;100%;Alteromonadaceae;
100%;Aliiglaciecola;78%
F5.8;+;Root;100%;Bacteria;100%;"Proteobacteria";
100%;Gammaproteobacteria;100%;Alteromonadales;100%;Alteromonadaceae;
100%;Aliiglaciecola;78%
F5.23;+;Root;100%;Bacteria;100%;"Proteobacteria";
100%;Gammaproteobacteria;100%;Alteromonadales;100%;Alteromonadaceae;
100%;Aliiglaciecola;78%
F5.5;+;Root;100%;Bacteria;100%;"Bacteroidetes";100%;Flavobacteriia;
99%;"Flavobacteriales";99%;Flavobacteriaceae;99%;Aureicoccus;34%
F5.32;+;Root;100%;Bacteria;100%;"Bacteroidetes";100%;Flavobacteriia;
99%;"Flavobacteriales";99%;Flavobacteriaceae;99%;Aureicoccus;34%
