# Supplementary material for: Impact of Ocean Acidification on the Intestinal Microbiota of the Marine Sea Bream (Sparus aurata L.)
Source: Front Physiol. 2019 Nov 28;10:1446. doi: 10.3389/fphys.2019.01446 (PMC6893888; doi:10.3389/fphys.2019.01446)
Supplement: Supplementary file 7 [file Data_Sheet_5.PDF]

Classifier: RDP Naive Bayesian rRNA Classifier Version 2.11, September 2015
Taxonomical Hierarchy: RDP 16S rRNA training set 16
Query File: F13-F16 GenBank.fas
Submit Date: Wed Jul 10 07:34:42 EDT 2019
Confidence threshold (for classification to Root ONLY): 95%
Symbol +/- indicates predicted sequence orientation

2F13.1_;+;Root;100%;Bacteria;100%;"Proteobacteria";100%;Alphaproteobacteria;100%;Rhodobacterales;100%;Rhodobacteraceae;100%;Roseovarius;56%
B2F13.17;+;Root;100%;Bacteria;100%;"Proteobacteria";100%;Betaproteobacteria;100%;Burkholderiales;100%;Oxalobacteraceae;100%;Herbaspirillum;100%
B2F13.18;+;Root;100%;Bacteria;100%;"Bacteroidetes";100%;Flavobacteriia;100%;"Flavobacteriales";100%;Flavobacteriaceae;100%;Gaetbulibacter;12%
BF13.18;+;Root;100%;Bacteria;100%;"Bacteroidetes";100%;Flavobacteriia;100%;"Flavobacteriales";100%;Flavobacteriaceae;100%;Gaetbulibacter;12%
BF13.20;+;Root;100%;Bacteria;100%;"Bacteroidetes";100%;Flavobacteriia;100%;"Flavobacteriales";100%;Flavobacteriaceae;100%;Gaetbulibacter;12%
BF13.25;+;Root;100%;Bacteria;100%;"Bacteroidetes";100%;Flavobacteriia;100%;"Flavobacteriales";100%;Flavobacteriaceae;100%;Gaetbulibacter;12%
BF13.28;+;Root;100%;Bacteria;100%;"Bacteroidetes";100%;Flavobacteriia;100%;"Flavobacteriales";100%;Flavobacteriaceae;100%;Gaetbulibacter;12%
B2F13.33;+;Root;100%;Bacteria;100%;"Bacteroidetes";100%;Flavobacteriia;100%;"Flavobacteriales";100%;Flavobacteriaceae;100%;Gaetbulibacter;12%
B2F13.20;+;Root;100%;Bacteria;100%;"Proteobacteria";100%;Alphaproteobacteria;98%;SAR11;91%;Candidatus Pelagibacter;91%
B2F13.25;+;Root;100%;Bacteria;100%;"Proteobacteria";100%;Alphaproteobacteria;100%;SAR11;100%;Candidatus Pelagibacter;100%
B2F13.27;+;Root;100%;Bacteria;100%;"Proteobacteria";100%;Gammaproteobacteria;100%;"Vibrionales";100%;Vibrionaceae;100%;Vibrio;46%
BF13.27;+;Root;100%;Bacteria;100%;"Proteobacteria";100%;Gammaproteobacteria;100%;"Vibrionales";100%;Vibrionaceae;100%;Vibrio;56%
BF13.32;+;Root;100%;Bacteria;100%;"Proteobacteria";100%;Gammaproteobacteria;100%;"Vibrionales";100%;Vibrionaceae;100%;Lucibacterium;61%
F13.3;+;Root;100%;Bacteria;100%;"Bacteroidetes";100%;Flavobacteriia;100%;"Flavobacteriales";100%;Flavobacteriaceae;78%;Zunongwangia;27%
F13.31;+;Root;100%;Bacteria;100%;"Bacteroidetes";100%;Flavobacteriia;100%;"Flavobacteriales";100%;Flavobacteriaceae;78%;Zunongwangia;27%
2F13.7;+;Root;100%;Bacteria;100%;"Proteobacteria";100%;Gammaproteobacteria;100%;"Vibrionales";100%;Vibrionaceae;100%;Vibrio;100%

2F13.8;+;Root;100%;Bacteria;100%;"Proteobacteria";
100%;Gammaproteobacteria;100%;"Vibrionales";100%;Vibrionaceae;
100%;Vibrio;55%
2F13.27;+;Root;100%;Bacteria;100%;"Proteobacteria";
100%;Gammaproteobacteria;100%;"Vibrionales";100%;Vibrionaceae;
100%;Vibrio;48%
2F13.16;+;Root;100%;Bacteria;100%;"Proteobacteria";
100%;Gammaproteobacteria;100%;"Vibrionales";100%;Vibrionaceae;
100%;Vibrio;100%
B2F13.35;+;Root;100%;Bacteria;100%;"Bacteroidetes";
100%;Flavobacteriia;99%;"Flavobacteriales";99%;Flavobacteriaceae;
78%;Kordia;17%
BF13.22;+;Root;100%;Bacteria;100%;"Bacteroidetes";
100%;Flavobacteriia;100%;"Flavobacteriales";100%;Flavobacteriaceae;
78%;Zunongwangia;27%
BF13.15;+;Root;100%;Bacteria;100%;"Bacteroidetes";
100%;Flavobacteriia;100%;"Flavobacteriales";100%;Flavobacteriaceae;
87%;Zunongwangia;30%
BF13.33;+;Root;100%;Bacteria;100%;"Verrucomicrobia";
100%;Verrucomicrobiae;100%;Verrucomicrobiales;
100%;Verrucomicrobiaceae;99%;Persicirhabdus;94%
B2F13.4;+;Root;100%;Bacteria;100%;"Verrucomicrobia";
100%;Verrucomicrobiae;100%;Verrucomicrobiales;
100%;Verrucomicrobiaceae;99%;Persicirhabdus;94%
B2F13.9;+;Root;100%;Bacteria;100%;"Verrucomicrobia";
100%;Verrucomicrobiae;100%;Verrucomicrobiales;
100%;Verrucomicrobiaceae;99%;Persicirhabdus;94%
F13.7;+;Root;100%;Bacteria;100%;"Actinobacteria";
100%;Actinobacteria;100%;Actinobacteridae;100%;Actinomycetales;
100%;Micrococcineae;100%;Microbacteriaceae;100%;Rhodoluna;49%
F13.9;+;Root;100%;Bacteria;100%;"Actinobacteria";
100%;Actinobacteria;100%;Actinobacteridae;100%;Actinomycetales;
100%;Propionibacterineae;100%;Propionibacteriaceae;
100%;Propionibacterium;100%
F13.2;+;Root;100%;Bacteria;100%;"Proteobacteria";
100%;Gammaproteobacteria;100%;Alteromonadales;100%;Alteromonadaceae;
100%;Aliiglaciecola;78%
F13.12;+;Root;100%;Bacteria;100%;"Proteobacteria";
100%;Gammaproteobacteria;100%;Alteromonadales;100%;Alteromonadaceae;
100%;Aliiglaciecola;78%
BF13.35;+;Root;100%;Bacteria;100%;"Proteobacteria";
100%;Gammaproteobacteria;100%;Alteromonadales;100%;Alteromonadaceae;
100%;Aliiglaciecola;78%
