# Supplementary material for: Impact of Ocean Acidification on the Intestinal Microbiota of the Marine Sea Bream (Sparus aurata L.)
Source: Front Physiol. 2019 Nov 28;10:1446. doi: 10.3389/fphys.2019.01446 (PMC6893888; doi:10.3389/fphys.2019.01446)
Supplement: Supplementary file 8 [file Data_Sheet_6.PDF]

Classifier: RDP Naive Bayesian rRNA Classifier Version 2.11, September 2015
Taxonomical Hierarchy: RDP 16S rRNA training set 16
Query File: F21-F24 GenBank VR2.fas
Submit Date: Wed Jul 10 07:22:14 EDT 2019
Confidence threshold (for classification to Root ONLY): 95%
Symbol +/- indicates predicted sequence orientation

F21.52_;+;Root;100%;Bacteria;100%;"Proteobacteria";100%;Gammaproteobacteria;100%;"Vibrionales";100%;Vibrionaceae;100%;Lucibacterium;68%
F21.25_;+;Root;100%;Bacteria;100%;"Proteobacteria";100%;Gammaproteobacteria;100%;"Vibrionales";100%;Vibrionaceae;100%;Lucibacterium;68%
F21.29;+;Root;100%;Bacteria;100%;"Proteobacteria";100%;Gammaproteobacteria;100%;"Vibrionales";100%;Vibrionaceae;100%;Vibrio;97%
F21.54_;+;Root;100%;Bacteria;100%;"Proteobacteria";100%;Gammaproteobacteria;100%;"Vibrionales";100%;Vibrionaceae;100%;Vibrio;100%
F21.44_;+;Root;100%;Bacteria;100%;"Proteobacteria";100%;Gammaproteobacteria;100%;"Vibrionales";100%;Vibrionaceae;100%;Vibrio;100%
F21.55;+;Root;100%;Bacteria;100%;"Proteobacteria";100%;Gammaproteobacteria;100%;"Vibrionales";100%;Vibrionaceae;100%;Vibrio;97%
F21.10_;+;Root;100%;Bacteria;100%;"Bacteroidetes";100%;Flavobacteriia;100%;"Flavobacteriales";100%;Flavobacteriaceae;100%;Spongiibacterium;83%
F21.45;+;Root;100%;Bacteria;100%;"Bacteroidetes";100%;Flavobacteriia;100%;"Flavobacteriales";100%;Flavobacteriaceae;100%;Spongiibacterium;75%
F21.20;+;Root;100%;Bacteria;100%;"Bacteroidetes";100%;Flavobacteriia;100%;"Flavobacteriales";100%;Flavobacteriaceae;100%;Spongiibacterium;75%
F21.14_;+;Root;100%;Bacteria;100%;"Proteobacteria";100%;Betaproteobacteria;100%;Burkholderiales;100%;Comamonadaceae;100%;Variovorax;100%
F21.15_;+;Root;100%;Bacteria;100%;"Planctomycetes";100%;Planctomycetia;100%;Planctomycetales;100%;Planctomycetaceae;100%;Gimesia;39%
F21.39;+;Root;100%;Bacteria;100%;"Bacteroidetes";100%;Flavobacteriia;100%;"Flavobacteriales";100%;Flavobacteriaceae;100%;Aquibacter;19%
F21.40_;+;Root;100%;Bacteria;100%;"Proteobacteria";100%;Alphaproteobacteria;100%;Rhodobacterales;100%;Rhodobacteraceae;100%;Jannaschia;86%
F21.46_;+;Root;100%;Bacteria;100%;"Proteobacteria";100%;Alphaproteobacteria;100%;Rhodobacterales;100%;Rhodobacteraceae;100%;Oceanicola;36%
F21.53_;+;Root;100%;Bacteria;100%;"Proteobacteria";100%;Alphaproteobacteria;100%;Rhodobacterales;100%;Rhodobacteraceae;100%;Roseovarius;61%
F21.48_;+;Root;100%;Bacteria;100%;"Proteobacteria";

100%;Alphaproteobacteria;100%;Rhodobacterales;100%;Rhodobacteraceae;
100%;Oceanicola;36%
F21.51;+;Root;100%;Bacteria;100%;"Proteobacteria";
100%;Alphaproteobacteria;100%;Rhodobacterales;99%;Rhodobacteraceae;
99%;Poseidonocella;25%
F21.49;+;Root;100%;Bacteria;100%;"Bacteroidetes";
100%;Sphingobacteriia;100%;"Sphingobacteriales";
100%;Chitinophagaceae;100%;Asinibacterium;95%
F21.58;+;Root;100%;Bacteria;100%;"Bacteroidetes";
100%;Sphingobacteriia;100%;"Sphingobacteriales";
100%;Chitinophagaceae;100%;Asinibacterium;96%
F21.50_;+;Root;100%;Bacteria;100%;"Bacteroidetes";
100%;Sphingobacteriia;96%;"Sphingobacteriales";96%;"Saprospiraceae";
96%;Haliscomenobacter;28%
F21.56_;+;Root;100%;Bacteria;100%;"Actinobacteria";
100%;Actinobacteria;100%;Actinobacteridae;100%;Actinomycetales;
100%;Micrococcineae;100%;Microbacteriaceae;100%;Rhodoluna;49%
F21.57_;+;Root;100%;Bacteria;100%;"Verrucomicrobia";
100%;Verrucomicrobiae;100%;Verrucomicrobiales;
100%;Verrucomicrobiaceae;99%;Persicirhabdus;94%
F21.47_;+;Root;100%;Bacteria;100%;"Bacteroidetes";
100%;Sphingobacteriia;100%;"Sphingobacteriales";
100%;"Saprospiraceae";100%;Lewinella;99%
F21.33_;+;Root;100%;Bacteria;100%;"Proteobacteria";
100%;Alphaproteobacteria;100%;SAR11;100%;Candidatus Pelagibacter;
100%
F21.43_;+;Root;100%;Bacteria;100%;"Proteobacteria";
100%;Betaproteobacteria;100%;Burkholderiales;100%;Burkholderiaceae;
100%;Ralstonia;99%
F21.9_;+;Root;100%;Bacteria;100%;"Proteobacteria";
100%;Betaproteobacteria;100%;Burkholderiales;100%;Burkholderiaceae;
100%;Ralstonia;100%
F21.32;+;Root;100%;Bacteria;100%;"Proteobacteria";
100%;Gammaproteobacteria;100%;Alteromonadales;100%;Alteromonadaceae;
100%;Aliiglaciecola;78%
F21.43;+;Root;100%;Bacteria;100%;"Proteobacteria";
100%;Gammaproteobacteria;100%;Alteromonadales;100%;Alteromonadaceae;
100%;Aliiglaciecola;78%
F21.36_;+;Root;100%;Bacteria;100%;"Planctomycetes";
100%;Phycisphaerae;100%;Phycisphaerales;100%;Phycisphaeraceae;
100%;Phycisphaera;100%
F21.17_;+;Root;100%;Bacteria;100%;"Planctomycetes";
100%;Phycisphaerae;100%;Phycisphaerales;100%;Phycisphaeraceae;
100%;Phycisphaera;100%
F21.26_;+;Root;100%;Bacteria;100%;"Planctomycetes";
100%;Phycisphaerae;100%;Phycisphaerales;100%;Phycisphaeraceae;
100%;Phycisphaera;100%
