# Supplementary material for: Impact of Ocean Acidification on the Intestinal Microbiota of the Marine Sea Bream (Sparus aurata L.)
Source: Front Physiol. 2019 Nov 28;10:1446. doi: 10.3389/fphys.2019.01446 (PMC6893888; doi:10.3389/fphys.2019.01446)
Supplement: Supplementary file 9 [file Data_Sheet_7.PDF]

Supplementary Table 1. Outputs of PERMDISP and PERMANOVA. Statistically significant factors (*p* >0.05 for PERMDISP and P<0.05 for Monte Carlo) are indicated in bold.

PERMDISP	df	F	*p*
Deviations from centroid	1: 1<br>2: 4	1.076	**0.186**
Treatment	Size	Mean	SE
400	3	20.089	1.6163
1200	3	22.582	1.7787
Pairwise comparisons		t	*p*
(400, 1200)		1.0373	**0.202**
Treatment	Size	Mean	SE
400	3	20.089	1.6163
1200	3	22.582	1.7787

PERMANOVA	df	Pseudo F	p	Unique perms	P (Monte Carlo)
CO_2	1	6.2236	0.098	10	**0.022**
Residual	4				
Total	5				
