# Supplementary material for: Impact of Ocean Acidification on the Intestinal Microbiota of the Marine Sea Bream (Sparus aurata L.)
Source: Front Physiol. 2019 Nov 28;10:1446. doi: 10.3389/fphys.2019.01446 (PMC6893888; doi:10.3389/fphys.2019.01446)
Supplement: Supplementary file 10 [file Data_Sheet_8.PDF]

Supplementary Table 2. Averages of square root-transformed abundances (Av. Abund) in the two sample groups of Fig. 1 (groups from unconstrained divisive tree method: 400 µatm CO_2 (F1-4, F9-12, F17-F20) and 1200 µatm CO_2 (F5-F8, F13-F16, F21-F24)), and breakdown of average similarity (Av. Sim) within each group (**A** and **B**) and average dissimilarity (Av. Diss) between the two groups (**C**), into contributions from each OTU (bold). OTUs were ordered in decreasing contribution, until at least 70% of average similarity within each group or dissimilarity between the two groups was attained (see last column). Ratios Sim/SD and Diss./SD (also bold) identify consistent discriminators by dividing average similarity or average dissimilarity by its SD. The SIMPER (similarity percentages – OTUs contributions) One-Way analysis was based on the Bray-Curtis similarity.

A- Contributions from each OTU to similarity between samples from group 400 µatm CO_2 (average similarity: **65.24**)

OTU	Av.Abund	**Av.Sim**	**Sim/SD**	Contrib%	Cum.%
Propionibacteriaceae	2.14	**14.22**	**9.42**	21.80	21.80
Flavobacteriaceae	1.97	**13.32**	**7.32**	20.41	42.21
Streptococcaceae	1.61	**10.64**	**15.84**	16.31	58.52
Rhodobacteraceae	1.75	**10.20**	**2.01**	15.64	74.16

B- Contributions from each OUTU to similarity between samples from group 1200 µatm CO_2 (average similarity: **60.93**)

OTU	Av.Abund	**Av.Sim**	**Sim/SD**	Contrib%	Cum.%
Vibrionaceae	2.90	**18.17**	**6.15**	29.83	29.83
Alteromonadaceae	1.63	**11.05**	**4.16**	18.13	47.96
Rhodobacteraceae	1.75	**9.52**	**2.45**	15.62	63.58
SAR11	1.38	**8.32**	**3.01**	13.66	77.24

C- Contributions from each OUT to dissimilarity between samples from groups 400 µatm CO_2 and 1200 µatm CO_2 (average dissimilarity **60.97**)

OTU	Av.Abund	Av.Abund	**Av.Diss**	**Diss/SD**	Contrib%	Cum.%
Vibrionaceae	0.00	2.90	**10.88**	**3.58**	17.84	17.84
Propionibacteriaceae	2.14	0.33	**6.62**	**3.03**	10.86	28.70
Alteromonadaceae	0.00	1.63	**6.06**	**5.21**	9.94	38.64
Streptococcaceae	1.61	0.00	**5.91**	**6.16**	9.69	48.33
SAR11	1.08	1.38	**3.29**	**1.57**	5.40	53.73
Verrucomicrobiaceae	0.00	0.91	**3.26**	**1.15**	5.35	59.08
Oxalobacteraceae	1.00	0.33	**3.20**	**1.17**	5.25	64.33
Burkholderiaceae	0.88	0.30	**2.86**	**1.24**	4.68	69.02
Flavobacteriaceae	1.97	1.43	**2.55**	**1.48**	4.17	73.19
